# PREPARING OLDER ADULTS FOR REMOTE EMPLOYMENT: OPPORTUNITIES AND CHALLENGES

**DOI:** 10.1093/geroni/igac059.2487

**Published:** 2022-12-20

**Authors:** Setarreh Massihzadegan, Shayna Gleason, Jan Mutchler, Caitlin Coyle

**Affiliations:** UMASS Boston, Cambridge, Massachusetts, United States; University of Massachusetts Boston, Boston, Massachusetts, United States; University of Massachusetts Boston, Boston, Massachusetts, United States; University of Massachusetts Boston, Boston, Massachusetts, United States

## Abstract

As Americans live longer, many are finding they need or want to remain longer in the workforce. When the COVID-19 pandemic transitioned much of the U.S. workforce into temporary or permanent remote employment, many older job seekers were left behind, wanting to compete in the ever-more technology-based job market but often without the requisite skills to do so. The present study evaluated a workforce training program (funded by a Department of Labor demonstration grant) that trained low-income workers over the age of 55 for remote employment. Approximately 60 older adults were trained across three 20-week cohorts. Our data sources included biweekly participant surveys, typing speed and Microsoft Office skill assessments, exit interviews with program “drop-outs,” focus groups, training observations, data from participant applications, and instructor assessments of each participant’s level of “job readiness” at the end of the program. Results revealed that participants had acute financial need for employment, a keen interest in working remotely, and a wide range of employment experiences and past job stability. Many of their career trajectories and workplace needs were affected by COVID-19. Results also showed promising improvements in participants’ technology skills and confidence in their ability to conduct a job search over the course of the program. The need for greater connectivity between participants and employers was identified as an area for improvement for the program. The results of this study contribute to the literature on workforce development by exploring how training programs might better prepare older adults for an increasingly remote job market.

